# Prevalence and characteristics of concomitant septic and crystal-induced arthritis: A hospital database and literature review

**DOI:** 10.1515/rir-2025-0021

**Published:** 2025-10-04

**Authors:** Kittikorn Duangkum, Pattawee Saengmongkonpipat, Pimchanok Tantiwong, Yada Siriphannon, Thida Phungtaharn

**Affiliations:** Division of Rheumatology, Department of Medicine, Khon Kaen Hospital, Ministry of Public Health, Khon Kaen, Thailand

**Keywords:** Concomitant septic and crystal-induced arthritis, coexistence of septic and crystal-induced arthritis

## Abstract

**Background and Objectives:**

Concomitant septic and crystal-induced arthritis is a rare condition. Failure to diagnose this condition can result in significant harm to the patient. This study aims to investigate the prevalence and characteristics of concomitant septic and crystal-induced arthritis.

**Methods:**

A retrospective study included patients diagnosed with concomitant septic and crystal-induced arthritis confirmed by positive bacterial culture and intracellular crystals in synovial fluid of the same joint, from January 1, 2015, to July 31 ,2024.

**Results:**

A total of 45 cases were defined as having the prevalence of concomitant septic and crystal-induced arthritis among patients with crystal-induced arthritis of 4% (45/1116). Demographic characteristics showed male predominance (73.3%) with a mean ± SD age of 62.8 ± 14.4 years. Acute monoarthritis (66.7%, *n* = 30), which primarily affected the knee (68.9%, *n* = 31), was the most common presentation. Fever was present in 95.6% of cases. The median synovial white blood cell (WBC) count was 61, 478 cells/μL (interquartile range: 33, 600–131, 030). The mean ± SD C-reactive protein (CRP) level was 215 ± 96.7 mg/L. Monosodium urate crystals were found in 80% (*n* = 36) of the cases. The predominant bacteria were Staphylococcus (48.9%, *n* = 22), with Methicillin-sensitive *Staphylococcus aureus* (MSSA) being the most common (28.9%, *n* = 13), followed by *Streptococcus dysgalactiae* (15.6%, *n* = 7) and gram-negative bacilli (15.6%, *n* = 7). The mortality rate was 15.6% (*n* = 7).

**Conclusion:**

The prevalence of concomitant septic and crystal-induced arthritis was 4% among patients with crystal-induced arthritis, especially among those with acute fever and high synovial WBC counts. The chance of concomitant septic and crystal-induced arthritis is very low in cases with synovial WBC < 12,000 cells/μL.

## Introduction

Crystal-induced arthritis is a common form of arthritis encountered in clinical practice, with gouty arthritis being the most prevalent. Its prevalence ranges from 0.9%–5.2%, with higher rates observed in Western countries.^[1,2]^ The gold standard for diagnosing gout is the detection of intracellular monosodium urate (MSU) crystals in synovial fluid.^[3]^ Calcium pyrophosphate dihydrate crystal deposition disease (CPPD) represents one of the most prevalent forms of crystal-induced arthritis, and is diagnosed by the detection of calcium pyrophosphate (CPP) crystals.^[4]^ The exact prevalence of CPPD is unknown due to there being a variety of clinical subtypes, including asymptomatic chondrocalcinosis; however, the incidence increases with age. One study in the UK showed that the prevalence of knee CPPD was 7%–10% in patients > 60 years of age.^[5]^ Septic arthritis occurs less frequently than crystal-induced arthritis; however, it is associated with a higher mortality rate of 11.5%.^[6]^ As a rheumatological emergency, this condition exhibits an incidence rate of approximately 2–10 cases per 100,000 person-years in the general population. In particular, this rate increases significantly to 30–100 cases per 100,000 person-years among individuals with certain risk factors, including older adults, immunocompromised hosts, patients with rheumatoid arthritis (RA), and those with prosthetic joints.^[7, 8, 9, 10]^

Diagnosis of septic arthritis can be challenging due to its symptoms and laboratory findings, such as acute monoarthritis or oligoarthritis with or without fever, high synovial fluid white blood cell (WBC) count, and leukocytosis, which are symptoms similar to those observed in crystal-induced arthritis.^[11]^ Delayed diagnosis and treatment of septic arthritis can lead to joint damage and increased mortality, significantly impacting both short- and long-term quality of life.^[12]^

Differentiating between septic arthritis and crystal-induced arthritis presents a notable challenge, particularly when patients with existing crystal-induced arthritis may also develop concomitant septic arthritis.^[11,13,14]^ The detection of crystals in synovial fluid does not exclude the possibility of concomitant septic and crystal-induced arthritis.^[11,13, 14, 15]^

Research surrounding concomitant septic and crystal-induced arthritis remains sparse, underscoring our motivation for this study. In this study, we aimed to investigate the prevalence and characteristics of concomitant septic and crystal-induced arthritis.

## Materials and Methods

### Study Design and Study Populations

We conducted a retrospective study at Khon Kaen Hospital, a tertiary care center in the northeastern region of Thailand, focusing on patients with concomitant septic arthritis and crystal-induced arthritis between January 1, 2015 and July 31, 2024.

All patients who underwent synovial fluid analysis during the study period were included. Septic arthritis was defined as positive synovial fluid bacterial cultures and crystal-induced arthritis was identified by the presence of at least one intracellular crystal in the synovial fluid visualized under light or polarized light microscopy. Concomitant septic and crystal-induced arthritis indicates the simultaneous occurrence of both septic and crystal-induced arthritis in the same joint.

### Study Processes

Our study consisted of three distinct steps.

First, eligible patients were selected from three data sources: I) the hospital’s electronic database, using the International Classification of Diseases 10th revision (ICD-10) codes M00, M10, and M11 for pyogenic arthritis, gout, and other crystal-induced arthropathies, respectively. II) The hospital laboratory database contained information regarding individuals who had undergone synovial fluid analysis. III) The Division of Rheumatology Service Database containing the results of light or polarized light microscopy examinations.

In the second step, septic and crystal-induced arthritis cases were identified from three databases for further analysis.

Finally, in the third step, we conducted a detailed analysis to determine the prevalence and characteristics of concomitant septic and crystal-induced arthritis.

This study was approved by the Institutional Review Board of the Khon Kaen Hospital (protocol code: KEXP65061).

### Statistical Analyses

Statistical analyses were performed using STATA software (Stata Corp, v. 13). Descriptive statistics were used based on data characteristics. Categorical variables such as sex, underlying disease, joint distribution, and mortality are presented as numbers and percentages. Continuous variables, including age, duration of pain, WBC count, and C-reactive protein (CRP) levels, were expressed as means with standard deviation (SD) or medians with interquartile ranges (IQR), depending on the distribution of the data.

## Results

**Figure 1:**
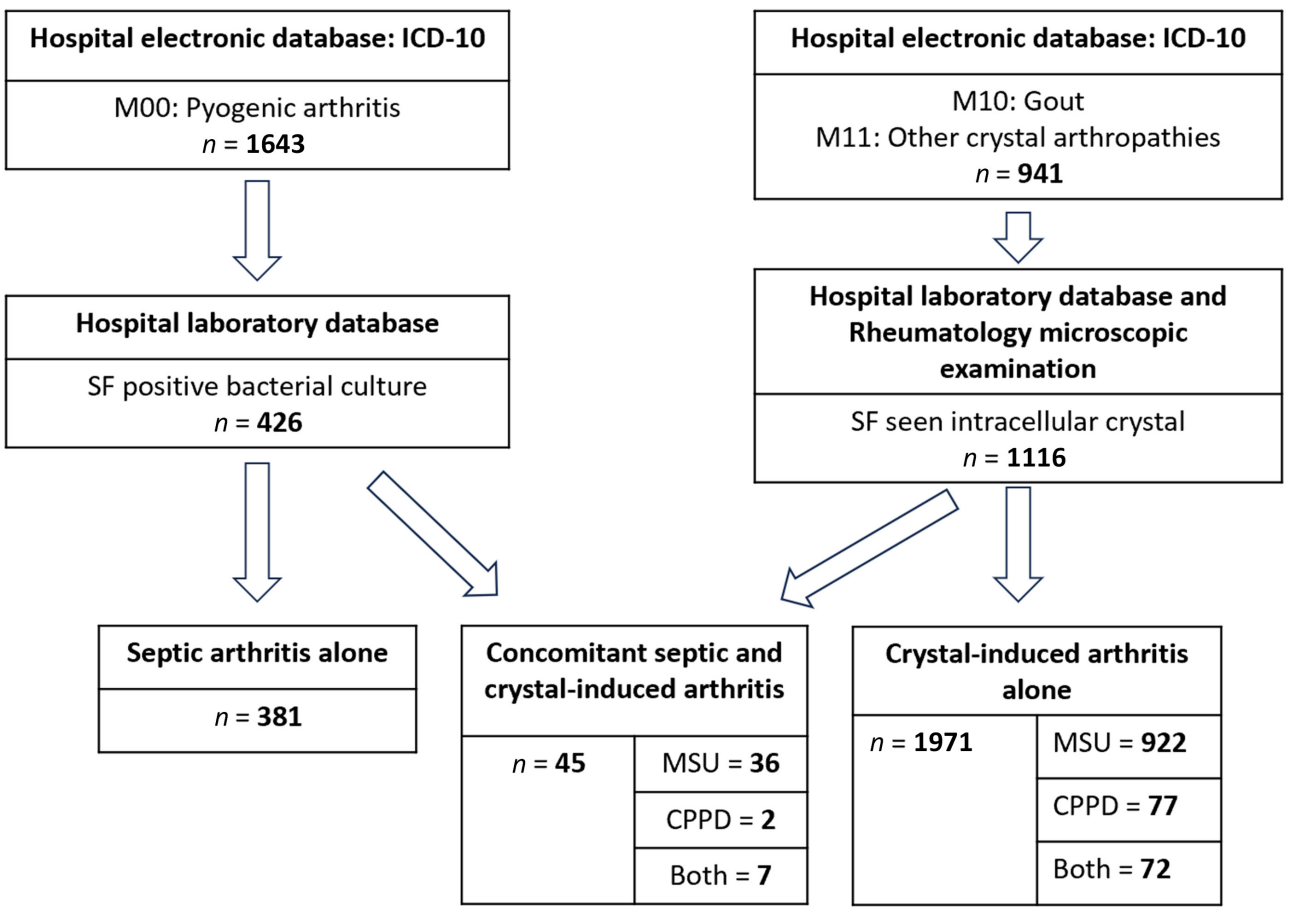
Patients enrollment. CPPD, calcium pyrophosphate dihydrate; ICD-10, International Classification of Diseases 10th Revision; MSUC, monosodium urate crystal; SF, synovial fluid.

Initially, 2, 584 individuals were selected from the hospital’s electronic database based on the ICD-10 codes M00, M10, and M11. Subsequently, 426 patients with septic arthritis and 1116 with crystal-induced arthritis were identified from the hospital laboratory database. Among them, 50 patients initially showed positive findings for synovial fluid bacterial cultures and intracellular crystals; however, after excluding five patients due to probable contamination of the bacterial culture, 45 patients with concomitant septic and crystal-induced arthritis remained for inclusion in the study, as illustrated in Figure 1.

### Prevalence of Concomitant Septic and Crystal-induced Arthritis

The prevalence of concomitant septic and crystal-induced arthritis was 4% (45/1116).

### Patient Baseline Clinical Characteristics

Table 1 summarizes the patient baseline characteristics, revealing that the majority of patients were male (73.3%) with a mean age (± SD) of 62.8 ± 14.4 years. Pre-existing gout was the most prevalent among underlying diseases (53.3%), with other conditions detailed in Table 1. The characteristics of each patient are presented in Table 2

**Table 1:** Patient baseline characteristics

Characteristics	Percent (Number)
Number of patients	45
Age, mean (SD)-years	62.8 (14.4)
Male sex	73.3 (33)
Duration of joint pain, median (IQR)-days*	3 (2–7)
Fever	95.6 (43)
Prior crystal attack	53.3 (24)
Number of joints	
Monoarthritis	66.7 (30)
Oligoarthritis	31.1 (14)
Polyarthritis	2.2 (1)
Joint involvement	
Knee	68.9 (31)
Ankle	28.9 (13)
Wrist	15.6 (7)
Others; elbow, hip, shoulder	13.3 (6)
SF WBC, median (IQR) [range]- cells/μL	61, 478 (33,600–131,030) [12,095–463,220]
SF WBC	
≤ 20, 000 cells/μL	13.2 (5/38)
20, 001–50, 000 cells/μL	26.3 (10/38)
50, 001–100, 000 cells/μL	26.3 (10/38)
> 100, 000 cells/μL	34.2 (13/38)
SF PMN, mean (SD)-%	85.9 (12.6)
Blood WBC, mean (SD)- cells/μL	16, 046 (8702)
CRP, mean (SD) [range]-mg/L	215 (96.7) [82.2–379.8]
SF gram stain positive	35.6 (16)
GPC	39.5 (15/38)
GNB	14.3 (1/7)
SF culture	
GPC	84.4 (38)
Staphylococcus	48.9 (22)
MSSA	28.9 (13)
MRSA	2.2 (1)
Streptococcus	31.1 (14)
*Streptococcus dysgalactiae*	15.6 (7)
Streptococcus agalactiae	11.1 (5)
GNB	15.6 (7)
SF crystal	
MSU	80 (36)
CPPD	4.4 (2)
Both	15.6 (7)
Hemoculture	15.6 (7)
GPC	13.2 (5/38)
GNB	28.6 (2/7)
Underlying disease before admission	
Pre-existing gout	53.3 (24)
Hypertension	48.9 (22)
Chronic kidney disease	37.8 (17)
Diabetes mellitus	22.2 (10)
Cirrhosis	13.3 (6)
Rheumatoid arthritis	11.1 (5)
Osteoarthritis	6.7 (3)
Surgical drainage	60 (27)
Outcome	
Death	15.6 (7)
Recovery	84.4 (38)

CPPD: Calcium pyrophosphate dihydrate deposition disease, GNB: C-reactive protein (CRP), GPC: gram-negative bacilli, gram-positive cocci, IQR: interquartile range, MRSA: methicillin-resistant *Staphylococcus aureus*, MSSA: methicillin-susceptible Staphylococcus aureus, mg/L: milligrams per liter, MSU: monosodium urate crystal, PMN: polymorphonuclear leukocyte, SD: standard deviation, SF: synovial fluid, WBC: white blood cell. *Defined as the time from joint pain onset to the initial detection of either intra cellular crystal or microorganism (by culture) in the synovial fluid.

**Table 2:** Characteristics of each patient

No	Sex	Age	Fever	Onset (Day)	Joint	Underlying disease	SF - cells/WBC μL (%PMN)	Synovial fluid culture	Crystal	Positive HC	CRP (mg/L)
1	M	63	Yes	1	Ankle	Gout, AF, HT	141,900 (65)	MSSA	MSU	No	252.2
2	M	65	Yes	1	Ankle	Gout, AF, HT, MI	TNTC	MSSA	MSU	No	N/A
3	M	49	Yes	2	Ankle	Gout, HT	TNTC	MSSA	MSU	No	N/A
4	F	55	Yes	2	Knee	ITP, RA	338,000 (65)	MSSA	Both	No	N/A
5*	M	76	Yes	3	Knee	HT, CKD4	31,530 (46)	MSSA	MSU	No	N/A
6	M	44	Yes	4	Knee	-	463,220 (72)	MSSA	MSU	No	221
7	M	45	Yes	4	Ankle, Knee	Gout, ESRD, HT	33,338 (95)	MSSA	MSU	No	N/A
8	M	67	Yes	5	Wrist	CKD3	TNTC	MSSA	MSU	No	N/A
9	F	65	Yes	5	Knee	Cirrhosis, DM, HT	42,129 (89)	MSSA	MSU	No	N/A
10	M	23	Yes	7	Polyarthritis	Lamellar ichthyosis	55,550 (93)	MSSA	MSU	No	379.8
11	M	50	Yes	14	Knee	Gout, alcoholic cirrhosis	TNTC	MSSA	MSU	No	N/A
12	M	51	Yes	30	Knee	Gout, HT	110,400 (94)	MSSA	MSU	No	N/A
13*	M	79	Yes	2	Wrist Knee	OA knees	N/A	MSSA	Both	Yes	N/A
14	M	66	Yes	30	Knee	DM, cirrhosis, CVA, AF	61,254 (97)	MRSA	Both	Yes	N/A
15	F	61	Yes	1	Knee	DM, HT, Old CVA	186,840 (90)	*Streptococcus dysgalactiae*	CPPD	Yes	N/A
16	M	55	Yes	2	Knee	Gout	41,878 (95)	*Streptococcus dysgalactiae*	MSU	No	N/A
17	M	46	Yes	5	Knee	Gout	197,110 (90)	*Streptococcus dysgalactiae*	MSU	No	N/A
18*	F	80	Yes	2	Polyarthritis	Old CVA	171,830 (83)	*Streptococcus dysgalactiae*	MSU	No	N/A
19*	M	52	Yes	2	Knee	Gout, DM, alcoholic cirrhosis	75,330 (56)	*Streptococcus dysgalactiae*	MSU	Yes	N/A
20*	M	71	Yes	3	Knee	Gout, CKD4, DM, HT	43,015 (94)	*Streptococcus dysgalactiae*	MSU	No	N/A
21*	M	68	Yes	7	Knee, Ankle	Gout, alcoholic cirrhosis	14,483 (94)	*Streptococcus dysgalactiae*	MSU	No	N/A
22	M	76	Yes	1	Knee	Gout, CKD3, DM, HT, DLP	85,826 (95)	*Streptococcus agalactiae*	CPPD	No	N/A
23	M	74	Yes	3	Shoulder	HT	N/A	*Streptococcus agalactiae*	MSU	No	N/A
24	M	48	Yes	4	Knee	Epilepsy	158,705 (81)	*Streptococcus agalactiae*	MSU	No	226.9
25	M	49	Yes	7	Ankle	Gout, CKD2	131,030 (85)	*Streptococcus agalactiae*	MSU	Yes	N/A
26	M	36	Yes	9	Ankle	-	120,500 (92)	*Streptococcus agalactiae*	MSU	No	N/A
27	F	82	Yes	2	Wrist, 1st MTP	Gout, CKD3, DM, HT	65,420 (84)	*Streptococcus pyogenes*	MSU	No	177
28	M	59	Yes	3	Ankle	Gout, DM, HT, DLP	39,530 (96)	*Streptococcus pyogenes*	Both	No	N/A
29	F	73	Yes	30	Knee, Ankle	HT, DLP	66,980 (94)	*Staphylococcus haemolyticus*	Both	No	100
30	F	82	Yes	7	Knees	HT, OA knees	59,382 (94)	*Staphylococcus haemolyticus*	Both	No	N/A
31	F	69	No	N/A	Knee	Gout, RA, HT, OA knees	N/A	*Staphylococcus warneri*	MSU	No	N/A
32	M	70	Yes	10	Knees	Gout, CKD3, DM, HT	20,000 (79)	*Staphylococcus hominis*	Both	No	N/A
33	M	84	Yes	1	Wrist, Elbow	Gout, CKD3	101,200 (90)	*Staphylococcus epidermidis*	MSU	No	N/A
34	M	74	Yes	1	Knee, Ankle	RA, CKD3	47,050 (96)	CONS	MSU	No	N/A
35	M	42	Yes	1	Knee	Gout	77,660 (98)	CONS	MSU	No	N/A
36	M	56	Yes	1	Knee	Gout, ESRD	15,170 (76)	MRCONS	MSU	No	N/A
37	M	85	No	4	Knee	Gout, CKD3, BPH	29,450 (93)	*Enterococcus faecium*	MSU	No	282
38	F	83	Yes	5	Knees, Ankles	Gout	33,600 (90)	*Lactococcus garvieae*	MSU	No	N/A
39	M	64	Yes	N/A	Knee	Multiple myeloma, CKD3	31,873 (95)	*Escherichia coli*	MSU	No	N/A
40	M	52	Yes	14	Hip	MTB lymphadenitis	12,095 (91)	*Salmonella* group B and C	MSU	No	N/A
41	F	74	Yes	8	Knee	Gout, CKD4, RA, HT	61,702 (97)	*Salmonella* group D	MSU	No	82.2
42	M	66	Yes	1	Knee	ESRD, HT, DM	17,363 (89)	*Sphingomonas paucimobilis*	MSU	No	N/A
43	M	62	Yes	7	Ankle	Gout, HT	355,740 (80)	*Burkholderia pseudomallei*	MSU	Yes	N/A
44	F	55	Yes	1	Knee	-	193,280 (90)	*Acinetobacter baumannii*	MSU	Yes	N/A
45*	F	82	Yes	3	Knee, Wrist	CKD4, HT, Cirrhosis	51,000 (71)	*Acinetobacter baumannii*	MSU	Yes	N/A

*AF: Patient with dead. Atrial fibrillation, BPH: benign prostatic hyperplasia, CPPD: calcium pyrophosphate dihydrate deposition disease, CVA: cerebrovascular accident, CKD: chronic kidney disease, CONS: coagulase negative Staphylococcus, CRP: C-reactive protein, DM: diabetes mellitus, DLP: dyslipidemia, ESRD: end stage renal disease, F: female, HC: hemoculture, HT: hypertension, ITP: immune thrombocytopenia, M: male, MI: myocardial infarction, MRCONS: methicillin-resistant coagulase negative Staphylococci, MRSA: methicillin-resistant *Staphylococcus aureus*, MSSA: methicillin-susceptible *Staphylococcus aureus*, mg/L: milligrams per liter, MSU: monosodium urate crystal, MTB: Mycobacterium Tuberculosis, MTP: metatarsophalangeal joint, N/A: not available, OA: osteoarthritis, PMN: polymorphonuclear leukocyte, RA: rheumatoid arthritis, SD: standard deviation, SF: synovial fluid, TNTC: too numerous to count, WBC: white blood cell.

### Clinical Presentations

Fever was observed in 95.6% of patients. Most patients presented with acute monoarthritis (66.7%), followed by acute oligoarthritis (31.1%), with acute polyarthritis being uncommon (2.2%). The knee was the most commonly affected joint (68.9%). The median (IQR) duration from joint pain onset to the diagnosis of either septic or crystal-induced arthritis was 3 (2–7) days.

### Laboratory Findings

The median synovial WBC count (IQR) was 61,478 (33,600–131,030) cells/μL. The mean (± SD) blood WBC count was 16,046 ± 8702 cells/μL. Monosodium urate crystals were detected in 80% of cases, whereas CPP crystals were identified in 4.4% of cases. CRP results were available for only 17.8% of patients. For these patients, the mean (± SD) CRP level was 215 ± 96.7 mg/L, and no patient recorded a CRP level below 82.2 mg/L.

The predominant organism identified was Staphylococcus, found in 48.9% of cases. Within this group, methicillin-sensitive *Staphylococcus aureus* (MSSA) accounted for 28.9%. Streptococcus was identified in 31.1% of culture-positive patients. Among these, *Streptococcus dysgalactiae* accounted for 15.6%. Gram-negative bacilli were identified in 15.6% of culture-positive patients. Positive synovial Gram staining was observed in 35.6% of the culture-positive patients, whereas positive blood culture was observed in only 15.6% of patients.

### Management and Outcomes

Surgical treatment was performed in 60% of cases. The mortality rate was 15.6%.

## Discussion

Despite the rarity and limited research concerning concomitant septic and crystal-induced arthritis among patients with crystal-induced arthritis, our study identified a prevalence rate of 4%. Previous studies in the United States reported lower rates, ranging from 1.5%–1.8%,^[11,16]^ whereas Australia documented a higher rate of 5.2%.^[14]^

The majority of patients were male (73.3%), with a mean age of 62.8 years. Gout was the most common underlying condition, present in 53.3% of patients. Fever was present in 95.6% of patients. The most common joint presentation was acute monoarthritis (66.7%), with the knee being the most frequently affected joint (68.9%). The median duration of arthritis was 3 days. The median synovial fluid WBC count was 61,478 cells/μL. The majority of patients (86.8%) had synovial WBC counts exceeding 20,000 cells/μL. The mean CRP level was 215 mg/L, and none of the patients had a CRP level < 82.2 mg/L. The most common crystals detected were monosodium urate crystals (80%). Gram-positive cocci were the most frequently isolated organisms and were identified in 84.4% of culture-positive patients. Among these cases, Staphylococcus was the most common (48.9%), and gram-negative bacilli were present in 15.6% of culture-positive patients. Among patients with positive cultures, synovial Gram staining was positive in 35.6%, while blood cultures were positive in only 15.6%.

We conducted a literature review and compared our results to those of previous studies. Owing to the rarity of concomitant septic and crystal-induced arthritis, our findings consisted mainly of case reports and four case series as noted in Table 3.^[13, 14, 15,17]^

**Table 3:** Compare the data from literature review

Country, publish year	Taiwan, 2003^[13]^		Taiwan, 2009^[15]^		Australia, 2012^[14]^		Spain, 2019^[17]^		Thailand, 2024	
Year of study	1987–2001		1998–2008		2004–2009		1985–2015		2015–2024	
Primary objective	Assess clinical features and outcomes of concomitant gout and septic arthritis.		Assess characteristic features of patients with coexistence of gout and septic arthritis.		Identify frequency of coexistence of crystal and septic arthritis. To compare these with regard to SF microscopy, CRP, HC.		Assess the characteristics of concomitant septic and crystal-induced arthritis.		Study the prevalence and characteristics of patients with concomitant septic and crystal-induced arthritis.	
Prevalence of concomitant arthritis	N/A		N/A		5.2%		N/A		4%	
Number of patients	30		14		22		25		45	
Age, mean (SD)-years	52.8 (12.5)		63.7 (10.9)		76 (N/A)		67 (14)		62.8 (14.4)	
Septic arthritis diagnosis	Positive bacterial culture in synovial fluid		Positive bacterial culture in synovial fluid		Positive bacterial culture in synovial fluid		-Positive culture in synovial fluid (22/25) -Positive HC but negative SF culture (3/25)		Positive bacterial culture in synovial fluid	
Crystal induced arthritis diagnosis	Seen intracellular crystal in synovial fluid or intraarticular tophi		Seen intracellular crystal in synovial fluid		Seen intracellular crystal in synovial fluid		Deposition of microcrystals		Seen intracellular crystal in synovial fluid	
Type of crystal MSU, % (*n*)	100 (30)		100 (14)		41.9 (13)		68 (17)		80 (36)	
Calcium, % (*n*)	0		0		CPPD	58.1 (19)	CPPD 20 (5) HA 12 (3)		CPPD 4.4 (2)	
Both, % (*n*)	0		0		0		0		15.6 (7)	
Male sex, % (*n*)	86.7 (26)		92.9 (13)		77.3 (17)		68 (17)		73.3 (33)	
Duration, mean (SD)-days	6.5		6.5 (4.0)		N/A		14 (13)		6 (7.4)	
Fever, % (*n*)	66.7 (20)		71.4 (10)		N/A		48 (12)		95.6 (43)	
Number of joint, % (*n*)	Monoarthritis	90	Monoarthritis	14.3	N/A		Monoarthritis	92	Monoarthritis	66.7 (30)
	Oligoarthritis	10	Oligoarthritis	78.6			Oligoarthritis	8	Oligoarthritis	31.1 (14)
	Polyarthritis	-	Polyarthritis	7.1			Polyarthritis	-	Polyarthritis	2.2 (1)
Distribution of joint, % (*n*)	Knee	80	Ankle	78.6	N/A		Knee	48	Knee	68.9 (31)
	Ankle	20	Knee	57.1			MTP	12	Ankle	28.9
	Shoulder	3.3	Elbow	21.4			Hip	12	(13)	
	Wrist	3.3				Ankle		8	Wrist	15.6 (7)
							Shoulder	8	Elbow	6.7 (3)
									Hip 4.4 (2), shoulder 2.2 (1)	
WBC, mean (SD)- cells/μL	18,290 (11,059)		N/A		N/A		N/A		16,046 (8,702)	
CRP, mean (SD) [range]-mg/L	N/A		N/A		224 (N/A)		137.16 (138.83)		215 (96.7) [82.2–379.8]	
SF WBC, mean (SD)	59,470 (55,330)		44,102 (30,306)		N/A		23,057 (22,903)		99,535 (101,228)	
[range]- cells/μL	[3200–154,500]		[11,610–85,000]				[4000–75,000]		[12,095–463,220]	
SF PMN, %	95.7		93.3		N/A				85.9	
SF Bacterial culture										
GPC, % (*n*)	73.3 (22)		85.7 (12)		N/A		100 (17)		84.4 (38)	
Staphylococcus, % (*n*)	53.3 (16)		71.4 (10)				48 (12)		48.9 (22)	
MRSA, % (*n*)	23.3 (7)		7.1 (1)				12 (3)		2.2 (1)	
GNB, % (*n*)	30 (9)		14.2 (2)				0		15.6 (7)	
Positive SF gram stain, % (*n*)	56.3 (9/16)		71.4 (10/14)		54.5 (12/22)		N/A		35.6 (16)	
Positive HC, % (*n*)	36.7 (11/30)		50 (7/14)		43.8 (7/16)		N/A		15.6 (7)	
Comorbidities, % (*n*)	DM	16.7	Gout	92.2	DM	24	N/A		Gout	53.3 (24)
	Cirrhosis	6.7	CKD	78.6	CKD	16			HT	48.9 (22)
	Hemodialysis	3.3	DM	21.4	KT	16			CKD	37.8 (17)
	TKR	6.7			HT	8			DM	22.2 (10)
									Cirrhosis	13.3 (6)
Surgical management, %	Debridement	46.7	Debridement	35.7	N/A		Debridement	36	Debridement	60 (27)
	Amputation	3.3	Amputation	7.1						
Dead, % (*n*)	6.7 (2)		28.6 (4)		N/A		8 (2)		15.6 (7)	

AF: Atrial fibrillation, CPPD: calcium pyrophosphate dihydrate, CVA: cerebrovascular accident, CKD: chronic kidney disease, CRP: C-reactive protein, DM: diabetes mellitus, DLP: dyslipidemia, ESRD: end stage renal disease, HC: hemoculture, HA: hydroxyapatite, HT: hypertension, ITP: immune thrombocytopenia, KT: kidney transplantation, MTP: metatarsophalangeal joint, MTB: Mycobacterium Tuberculosis, mg/L: milligrams per liter, MRSA: methicillin-resistant *Staphylococcus aureus*, MSU: monosodium urate crystal, N/A: not available, OA: osteoarthritis, PMN: polymorphonuclear leukocyte, RA: rheumatoid arthritis, SD: standard deviation, SF: synovial fluid, TKR: total knee replacement, WBC: white blood cell.

Given the similarities in symptoms, such as acute monoarthritis or acute oligoarthritis with or without fever, and shared laboratory findings, such as elevated synovial fluid WBC counts and leukocytosis, diferentiating between septic arthritis and crystal-induced arthritis presents a significant challenge. This dificulty is further compounded in cases of concomitant septic arthritis and crystal-induced arthritis that occur in patients with crystal-induced arthritis. Even with the presence of crystals in synovial fluid, a diagnosis of concomitant septic arthritis and crystal-induced arthritis cannot be ruled out.^[11,13, 14, 15, 16, 17]^ Delayed diagnosis and treatment of septic arthritis can result in joint damage and increased morbidity and mortality. According to our literature review, the mortality rate associated with concomitant septic and crystal-induced arthritis ranges between 6.7%–28.6%.^[12, 13, 14, 15,17]^

Several studies have aimed to differentiate concomitant septic and crystal-induced arthritis from crystal-induced arthritis. Gram staining demonstrates high specificity and positive predictive value (PPV) in the diagnosis of septic arthritis.^[14]^ However, due to its low sensitivity, it produced a positive result in only 35.6% of the cases in our study. A synovial WBC count ≤ 10, 000 cells/μL and CRP level ≤ 100 mg/L were unlikely to indicate concomitant septic and crystal-induced arthritis, with negative predictive values (NPV) of 98.5% and 98.7%, respectively. In contrast, elevated synovial WBC counts > 10,000 cells/μL or increased CRP levels > 100 mg/L were suggestive of concomitant septic and crystal-induced arthritis, demonstrating a sensitivity of 86.4% and specificities of 48.3% and 54.6%, respectively.^[14]^ Compared to our findings, synovial WBC counts < 12,000 cells/μL and CRP levels < 80 mg/L were indicative of a lower likelihood of concomitant septic and crystal-induced arthritis. Elevated synovial WBC count > 85,000 cells/μL strongly indicates concomitant septic and crystal-induced arthritis with a specificity of 100%, although with a low PPV of 12.0%.^[16]^ This is consistent with our study, where 86.8% of patients had synovial WBC counts > 20,000 cells/μL, and 60.5% had synovial WBC > 50,000 cells/μL. Procalcitonin is a biological laboratory marker of bacterial infection that may be useful for diagnosing septic arthritis and acute osteomyelitis.^[18]^ A recent diagnostic study demonstrated that a procalcitonin level of ≥ 1.36 μg/L is highly specific for distinguishing septic arthritis from gouty arthritis, with a sensitivity of 70.4%, specificity of 99.5%, and a PPV of 97.4%. Conversely, a procalcitonin level of < 0.4 μg/L unlikely to be septic arthritis, with a sensitivity of 96.3%, specificity of 67.8%, and a NPV of 98.5%.^[19]^

Practitioner Points: In medical practice, we would like to suggest that the use of basic investigation to rule out concomitant septic and crystal-induced arthritis is easier than diagnosing such conditions, if synovial WBC < 12,000 cells/μL and CRP < 80 mg/L, or procalcitonin level < 0.4 ng/μL, the chance of concomitant septic and crystal-induced arthritis is very low. In contrast, if the synovial fluid Gram stain is positive, it has high specificity and PPV for the diagnosis of concomitant septic and crystal-induced arthritis. However, in typical cases where excluding this diagnosis is challenging, such as when synovial WBC > 20,000 cells/μL with fever or WBC > 85,000 cells/μL or procalcitonin level ≥ 1.36 ng/μL, it could be best to initially manage the condition as septic arthritis until further evidence allows a definitive exclusion of concomitant septic arthritis.

The strength of our study is that it encompasses the largest concomitant septic and crystal-induced arthritis population ever studied to date. One limitation of this study is that it was retrospective; it may lack some data, and the observed distribution of afected joints might be biased towards those more easily accessible for aspiration by physicians.

## Conclusions

The prevalence of concomitant septic and crystal-induced arthritis was 4% among patients with crystal-induced arthritis. Most patients presented with acute monoarthritis, fever, and elevated WBC counts in synovial fluid. The chance of concomitant septic and crystal-induced arthritis is very low in cases with synovial WBC counts < 12,000 cells/μL.
